# Diagnostic Markers for Nonspecific Inflammatory Bowel Diseases

**DOI:** 10.1155/2018/7451946

**Published:** 2018-06-11

**Authors:** Alicja Derkacz, Pawel Olczyk, Katarzyna Komosinska-Vassev

**Affiliations:** ^1^Department of Clinical Chemistry and Laboratory Diagnostics, School of Pharmacy and Division of Laboratory Medicine in Sosnowiec, Medical University of Silesia in Katowice, Jedności 8, 41-200 Sosnowiec, Poland; ^2^Department of Community Pharmacy, School of Pharmacy and Division of Laboratory Medicine in Sosnowiec, Medical University of Silesia in Katowice, Kasztanowa 3, 41-200 Sosnowiec, Poland

## Abstract

The nonspecific inflammatory bowel diseases (IBD) represent a heterogeneous group of chronic inflammatory disorders of the gastrointestinal tract, and Leśniowski-Crohn's disease (CD) and ulcerative colitis (UC) are among the two major clinical forms. Despite the great progress in understanding the pathogenesis of these diseases, their etiology remains unclear. Genetic, immune, and environmental factors are thought to play a key role. The correct diagnosis of nonspecific inflammatory bowel diseases as well as the determination of disease activity, risk stratification, and prediction of response to therapy still relies on a multidisciplinary approach based on clinical, laboratory, endoscopic, and histologic examination. However, considerable effort has been devoted to the development of an accurate panel of noninvasive biomarkers that have increased diagnostic sensitivity and specificity. Laboratory biomarkers useful in differentiating IBD with functional disorders and in evaluating disease activity, prognosis, and treatment selection for IBD are presented in this study.

## 1. Nonspecific Inflammatory Bowel Diseases—Introduction

Nonspecific inflammatory bowel diseases (IBD) constitute a group of diseases characterized by chronic inflammation of the digestive tract. Despite significant progress in understanding the pathogenesis of these disorders, their etiology remains unclear. For most of them, two clinical units are distinguished: Leśniowski-Crohn's disease (CD) and ulcerative colitis (UC). CD and UC possess a broad spectrum of clinical manifestations, and their heterogeneity has been underlined in many epidemiological surveys, concentrated over the identification of subgroups and phenotypes of these diseases. However, about 10–15% of IBD patients do not meet the criteria of UC or CD. Those cases are qualified as indeterminate colitis (IC) [1–4]. IBD also include sporadic diseases, such as Behcet's disease, collagenous colitis, microscopic enteritis, and eosinophilic enteritis. In the case of CD, chronic inflammation can be localized in every gastrointestinal tract segment and involves the full thickness of the intestinal wall. In UC, by contrast, mucose and submucose membranes of the large intestine are usually involved. Both diseases differ in the localization and size of the segment involved. There are also differences in clinical image, laboratory test results, and different characteristics of complications. The course and grade of disease activity depend on many factors, such as environmental influences, genetic features, changes in the intestinal microbiota ecosystem, and immune factors. Thanks to a quick diagnosis and applying an appropriate treatment, it is now possible to achieve the expected therapeutic results—the improvement of a patient's general condition and elimination or reduction of the risk of physical development delay (body weight and height deficit, delayed puberty) [5–8]. Correct diagnosis can be delayed by the lack of specific symptoms in the early stages of disease. IBD most often manifest with symptoms from the digestive tract, including abdominal pains of various intensities and locations, diarrhea (stools passes with pus and/or blood), nausea, bloating, flatulence, vomiting, and perianal changes. Moreover, systemic, generalised symptoms may occur, including a body weight deficit linked to absorption disorders and diarrhea. Growth retardation, unexplained fevers, fatigue, and exhaustion are very often the first signs the patients revealed. For IBD, extraintestinal symptoms may also be characteristic, such as arthralgia, joint inflammation, muscle pains, skin changes, oral lesions, kidney disease, ocular changes, and liver and bile duct disorders. The disease may lead to burdensome and often life-threatening complications, such as gastrointestinal bleedings, ileus, perforations, abscesses, fistulas, and toxic megacolon. In the course of the disease, the episodes of exacerbations and remissions are characteristic [9–11].

The etiology of CD is complex. At the moment, it is assumed that the disease develops under the influence of negative environmental determinants and genetic preconditions [12]. The contribution of environmental factors in the initiation of CD seems indisputable, as it is supported with the data from the research on migratory populations. Based on the analysis of the migrant population from the Caucasian region and South Asia to the UK (young-age immigration) in the 70s and 80s and analysis of the frequency of CD in this group, it has been confirmed that environmental factors play a role [13]. Differences in CD prevalence and the data indicating its significant growth in the last decades in some countries may point to the regional and population differences in exposure to environmental and/or genetic factors [14, 15]. Of major significance may be exposure to infections, dietary customs, hygiene, drugs, and vaccinations [16, 17].

Swedish research suggests that increased risk for CD may be connected to prenatal infections, especially with morbillivirus [18]. This was not confirmed by the UK research, where no correlation has been found between German measles and any other infection in pregnancy and increased risk of CD [19]. In some papers, a higher risk of CD has been emphasized among subjects, who suffered recurrent infections of upper airways and throat inflammations [20, 21]. Some other paper, however, did not support those findings [22]. In many papers, the role of bowel infection-triggering bacteria has been raised (i.e., *Mycobacterium*, *Campylobacter*, *Listeria*, *Escherichia*, *Salmonella*, *Clostridium*, *Yersinia*, and *Chlamydia*), as they directly lead to mucose membrane lesion. The indirect influence of toxins and other microbes (*Herpes*, *Rotaviruses*, and *Morbillivirus*) and fungi has been proposed [23–30]. Among those suffering from CD, microscopic stool examination revealed *Strongyloides stercoralis* and *Blastocystis hominis* [31]. Tapeworm infestation (common among children in developing countries) decreases the risk of CD [32], as it modifies the immune reactions towards bacteria and fungi [33]. It has been known that access to hot water and a separated bathroom in early childhood are associated to an increased risk of CD in later life, which means that better hygiene in childhood may predispose to CD [34].

The influence of breastfeeding, vaccinations, and appendectomy had also been analysed as potential contributors to the development of CD, yet positive results have not been obtained [35]. It is marked that the causes of the disease may also be linked to the usage of NSAIDs and oral contraception [36]. The potential cause may also lay in nutritional factors. It has been confirmed that a carbohydrate-rich diet (common in developed countries with a high incidence of CD) may increase the risk of CD [37]. Many of the protein antigens that mucosal cells meet may initiate or modify immune reactions in CD [38]. In 2003, a hypothesis has been raised, stating that compliance to CD depends on the polymorphism of xenobiotic-metabolizing enzymes (XME) [39], which refers to a risk connected with ingestion of fatty acids, vegetables, and fruits [40].

Among environmental factors, including familial ones affecting IBD development, smoking or exposure to tobacco smoke seems to be of special relevance. Smoking seems to have both positive and negative effect on IBD [41–45]. The influence of nicotine on Crohn's disease expression has been confirmed in the analyses of families with CD. In the European population, the incidence of smoking was 64% in families with CD [41]. Active and passive smokers have a higher risk of CD compared to nonsmokers and persons not exposed to tobacco smoke [42]. Independent studies have also shown that current and former smoking increases the risk of developing CD [44, 46]. Moreover, smoking has a negative impact on disease activity and the quality of life of patients with this disease entity [47]. On the other hand, tobacco smoking has been demonstrated to be protective against UC and after onset of the disease. Paradoxically, cigarette smoking might also improve the course of UC by decreasing the risk of colectomy [43]. The results of epidemiological studies indicate that among patients with UC, smoking habit is less common than among the average population outside of UC. It has also been demonstrated that the incidence of UC remains unchanged among the current smokers, but patients with UC are more susceptible to disease if they quit smoking [46]. The current state of knowledge confirms that the risk of developing UC is at the highest from 2 to 5 years after quitting and remains elevated for over 20 years [44]. Some studies reported that gender may influence the effect of tobacco smoking on UC. It has been found that current smoking delays disease onset in men but not in women [48]. As regard the link between the protective effect of active tobacco smoking on the severity of UC, some studies proved lower flare-up, hospitalization rates, and the need for oral steroids in smokers, compared with nonsmokers [48]. Some significance in the development of IBD may be from psychological factors [49]. The role of stress or compliant, “oversensitive and referent” psychics is underlined. Stress may play the role of a “trigger mechanism” changing neurotransmission and endocrine balances and modifying immune response [50]. The functional linkage of neuromodulators and neurotransmitters with their receptors, common to the digestive tract and nervous system, seems to be relevant. There is a suspicion that the neuropeptides (present in mucose-associated lymphoid tissues as well) may reverse phenotype Th1 or Th2, thus modifying inflammation and immune response [51]. To sum up, we can determine, regarding the present knowledge on the topic, that CD and UC, known as nonspecific inflammatory bowel diseases, are disorders the etiologic bases of which have not yet been fully determined. Microbial factors have been taken into consideration, as well as immune imbalances and genetic grounds. Racial concerns (Ashkenazi Jews, Caucasian race) and increased risk of disease among cosanguinated couples speak for the genetics. It is probable that equal parts are taken by immune and microbial factors. It is assumed that in people with genetically-determined sensitivity, an immune reaction against intestinal, nonpathologic microbiota takes place [52].

## 2. Diagnosis of Nonspecific Inflammatory Bowel Diseases (IBD)

Regarding lack of standards for diagnosis of IBD, the diagnostic process requires conjoint analysis of anamnesis, physical examination, and accessory tests. Endoscopic evaluation plays a key role with the biopsy of mucosa and subsequent histological examination of the specimen [53].

### 2.1. Anamnesis and Physical Examination

The diagnostic value of anamnesis and physical examination is minor. It results from the course of the disease and diversity symptoms. The main symptom of UC is increased frequency of bowel action and decreased stool consistency. The more blood in the stool, the more clinical manifestation resembles a typical image for UC. Pain in UC usually shows up in the left lower abdominal quadrant and is exacerbated before, yet alleviated after defecation [54]. In CD, diarrhea usually occurs less frequent and abdominal pains are often more likely. A typical syndrome of CD is pain in the lower right quadrant of the abdomen that intensifies after eating. The diagnosis of CD is usually made up when full-thickness transmural inflammation is observed with a segmental, discontinuous spread throughout the small and large intestines, usually excluding the rectum; it may be accompanied by fistulas. In CD, ulcerations are usually linear, deep, with elevated margins, and with a cobblestone pattern. Characteristic microscopic features of CD are neutrophilic and lymphocytic infiltrations, passing from the lamina propria into submucosa. Typical findings also include epitheloid granulomas composed of macrophages and lymphocytes, often ill circumscribed. Only lamina propria granulomas, without intestinal crypt lesions, are thought to confirm the diagnosis of CD [55]. Inflammation in UC usually spreads continuously, usually beginning and reaching its maximum in the rectum, and is characterized by fragile mucosa, blurred vessel web, and the presence of ulcerations in the spots of intensified inflammation. Continuous infiltration extends only to the mucosa, exclusively reaching deeper layers in the spots of vast ulceration. Microscopic findings include numerous, cell-abundant infiltrations in lamina propria, consisting not only of neutrophils, macrophages, and lymphocytes, but also of an increased number of eosinophils. In the mucosal cells, epithelium diminution of mucose-secreting cells is clearly visible. In intestinal crypts, one can see infiltrating neutrophils and so-called crypt microabscesses [36]. Progress in endoscopy techniques enables more advanced and precise evaluation. High hopes emerge with conjoining various diagnostic methods, that is, endosonography. An endoscopic capsule enables taking a look into the small bowel. Optic coherent tomography (OCT), penetrating 1.5 mm deep into the tissue layer, enables microscopic evaluation in vivo. Via OCT, it is possible to distinguish between UC and CD, by visualizing transmural inflammation, when colonoscopy and microscopic examination revealed no disturbances [33].

### 2.2. Laboratory Biomarkers

Laboratory tests in the diagnosis of IBD play an auxiliary role. They are useful mainly in differentiating IBD from functional disorders, detecting secondary nutritional deficiencies, and evaluating disease activity. Acute phase proteins, including C-reactive protein (CRP), despite being nonspecific for IBD, reflect inflammation intensity and help monitor the patient's condition [27, 56, 57]. Other routine blood tests, like FBC, ESR, and serum albumin have also been found useful in workup, but do not confirm nor differentiate IBD [14] (Table 1).

A supplement or alternative to the aforementioned may be a blood *test evaluating neutrophil activation*. This may reflect the process, the course of which starts with cellular-endothelial adhesion changes in mucosal microcirculation, facilitating transmigration of T and B lymphocytes from the vessels to the inflamed tissues, and thus aggregation of cells within the tissue, mastocytes, macrophages, eosinophils, and activated neutrophils is also included [29, 56].

The majority of studies emphasize on the cells responsible for specific immunity: lymphocytes and antigen presenting cells; less is known about the pathogenetic role of neutrophils in IBD. Microscopic examinations of tissues obtained from the patients with CD and UC exhibit the presence of neutrophils within inflammatory infiltrations [9]. In 90% of bowel epithelial cells in patients with IBD, a greater amount of *ENA-78 (epithelial neutrophil activating peptide)* has been shown [10, 21]. Activated by inflammatory stimulus (infection, trauma, and toxins), neutrophils secrete substances stored intracellularly, that is, proteolytic enzymes (leucocyte elastase) and numerous proteins of antibacterial function (calprotectin, lactoferrin). Blood or stool testing towards these substances is not routine, but potentially useful in the diagnosis of IBD as markers of neutrophil activation in the course of the inflammatory process.

*Human leucocytic elastase (HLE)* is the main protein secreted by activated neutrophils, ejected during exocytosis from azurophilic granules, and the main factor promoting tissue destruction in inflammatory diseases (e.g., acute respiratory distress syndrome (ARDS), lung emphysema, glomerulonephritis, or rheumatoid arthritis) [6]. 90% of HLE circulating in the bloodstream is bound to an endogenous inhibitor (*α*-1-antitrypsin; *α*1AT) irreversibly creating an enzyme-inhibitor complex. When in inflammatory focus, the inhibitor becomes inactivated itself by reactive oxygen species (ROS) released from activated neutrophils [10, 58]. That way, unbound elastase starts proteolytic degradation of connective tissue components such as elastin, collagen proteins, and proteoglycans [51]. Leucocyte elastase demonstrates many other functions: promoting neutrophil migration and adhesion, stimulating proinflammatory cytokines production, degrading phosphatidylserine receptors on macrophages, and decreasing neutrophil phagocytic ability [35]. Moreover, it has been shown that neutrophil-derived elastase has an influence over cell proliferation and may impair regenerative process in intestinal mucosa [1].

*Calprotectin* is a group of protein heterocomplexes: MRP-8/MRP-14 or S100A8/A9 (calcium-binding proteins, similar to migration-inhibiting factors: MRP-8 (S100A8) and MRP-14 (S100A9)). These proteins are expressed mainly in neutrophil and monocyte cytosols. Complex forming is calcium-dependent [1]. Calprotectin stands for 60% of the circulating neutrophil cytosolic proteins, and is also present in monocytes and macrophages as well as in the tissue eosinophils of the ileum. Peripheral blood monocytes expose calprotectin both intra- and extracellularly, but neutrophils only intracellularly. Calprotectin shows antibacterial, antifungal, immunomodulatory, and antiproliferative action. Moreover, it potentially is a chemotactic factor for neutrophils. Calprotectin concentration in serum increases in disorders with an increase in neutrophil action. Neutrophils possess the ability to transmigrate the intestinal wall; that way calprotectin may be present in the stool. It has been shown that calprotectin concentration markedly increases in bowel disorders, such as CD, UC, and colonic neoplasia [1]. Calprotectin may be of use when detecting inflammation within the bowel [1], helpful when diagnosing IBD. What is interesting is that elevated calprotectin concentration has been found in a healthy, first degree relative of a CD patient, which may suggest a potential application in determining the risk of CD development within relatives of the patient [2]. Nowadays, the attempts are made to introduce into clinical practice testing for calprotectin in stool as a routine. It has been demonstrated that *fecal calprotectin (FC)* concentration measurement is a highly sensitive and specific marker of intestinal disease differentiation between IBD and intestinal dysfunction in adults. Determination of fecal calprotectin is recommended in primary and secondary care in adults with recent gastrointestinal symptoms who are not suspected of cancer and in pediatric cases but only in supplementary care facilities [59].

The fecal calprotectin testing should be used in combination with other parameters including clinical, endoscopic, radiological, or histological. Such parameters allow for a comprehensive evaluation of the patient [60].

Fecal calprotectin has been used in many studies to predict a clinical relapse. Most of the studies looked at patients with CD and UC patients in clinical remission, measuring the initial fecal calprotectin concentration and observing them for at least 12 months to identify patients who had a clinical relapse. The mean fecal calprotectin concentration was compared between patients with relapses and no relapse. In most studies, a statistically higher initial fecal calprotectin concentration was found in CD patients and in patients with UC who later had relapses compared to patients without relapses. The results suggest that FC may be a better biomarker for colitis in patients with UC, because in adult patients with UC the test results were statistically significant in contrast to results in CD patients [61]. Fecal calprotectin proved its predictive value as a noninvasive marker of intestinal inflammation. However calprotectin is more suitable for predicting clinical relapse in ulcerative colitis than in Crohn's disease [62–64].

*Lactoferrin* is a glycoprotein transferrin produced mainly by the neutrophils. It takes part in nonspecific immune response thanks to its high affinity to iron and iron “capturing,” thus making iron inaccessible to bacteria. Another feature of lactoferrin is its resistance against proteolytic enzymes, which makes it possible to cross the digestive tract unchanged. Lactoferrin also plays an important role in modulating anti-inflammatory processes. Fecal lactoferrin increases significantly with bowel infiltration by neutrophils. The first papers on testing lactoferrin in the stool of IBD patients emerged after year 2000 [10, 58, 65, 66]. Like with calprotectin, attempts are made to use lactoferrin testing in the evaluation of patients with IBD. Up-to-date research confirms that testing lactoferrin in the stool may be of use in the diagnosis of CD and UC thanks to its high diagnostic sensitivity and specificity [10, 65].

Bowel mucosal cells, which underwent the influence of a traumatizing factor, for example, in the course of inflammation, liberate their intracellular constituents, like the *intestinal fatty acid binding protein (I-FABP)* bowel-specific protein binding long-chain fatty acids, bile acids, and retinoids. I-FABP is produced only by mature enterocytes of the small bowel, so it may be considered a specific marker of enterocyte lesion/necrosis. Human I-FABP makes up to 1-2% of the mature enterocyte cytosol proteins [6]. Still, little is known about the role of I-FABP in the diagnosis of IBD.

One of the sensitive markers of cellular immune response is *neopterine*. Testing for neopterine concentration in body fluids brings information of current state of immune response and may help predicting the progress of the disease. Neopterine release begins about three days before T-cells reaching proliferative maximum. Increase of neopterine biosynthesis is usually observed a week before specific antibodies to appear in the blood, thus neopterine is recommended as the early marker of inflammatory reaction [9]. Regarding this data, testing for serum neopterine may appear a useful tool to assess clinical activity of IBD [9].

In diagnosis and differentiating of IBD progressively more attention is being paid to *antibody testing*. It is supposed that they might be markers indicating immune disorders, like cross-reactions with environmental-derived antigens. Usefulness of many serological markers has been analysed in differentiating, activity determining, prognosis, and treatment selection for IBD. Hope emerges when considering anti neutrophil cytoplasmic antibodies *(ANCA)* and anti-*Saccharomyces cerevisiae* autoantibodies *(ASCA)*. First, ANCA were detected in patients suffering from various types of vasculitis. The term atypical pANCA (with perinuclear luminescence in method of indirect immunofluorescence) determines antibodies nonreactive towards myeloperoxidase, which can be detected mainly among patients with IBD and primary sclerosing cholangitis – PSC. pANCA occur among 50–80% of patients with UC and only among 5–10% with CD. Moreover, it has been observed that pANCA also occur among about 15% first-degree relatives of UC patients [9]. Nonpathogenic fungi *S. cerevisiae*, present in many foods, do not seems to be etiological factor of CD. Among the patients with IBD, ASCA are more characteristic for CD – they occur in 50–70% of CD cases, but only in 5–15% of UC cases [1]. These antibodies do not, however, occur among all IBD patients, for that sake new autoantibodies or antibacterial antibodies, potentially useful in differential diagnosing, are the subject of research. There is also a need to work out tests identifying patient subgroups, in which treatment against pathogenic microorganisms may be of a particular use. Many such antibodies, also useful in differential diagnosis, have already been discovered, for example, *anti-OmpC* (anti-*E. coli* C-cell wall canal protein), anti-mycobacterial histone-resembling protein *(Hup-B)*, anti I2-sequence of *Pseudomonas fluorescens (anti-I2)*, and anti-bacterial peptidoglycan *(ALCA, AMCA, and ACCA)*. Antibodies versus self-cells and tissues had also been described, including: anti-goblet cell antibodies *(GAB)*, characteristic for UC, and versus antigens of exocrine pancreas *(PAB)*, more likely to be found in CD, *catepsin G-reacting* or flagellin CBir1-reacting *(anti-CBir1 flagellin)* [10]. pANCA, ASCA, I2, and OmpC altogether can be found in 80% of patients with CD, only 20% of patients remain seronegative. It has been also reported that the presence of several serologic markers at diagnosis of CD is associated with the development of more complicated disease. It is discussed whether broadening of antibody tests spectrum may become crucial for differential diagnosis of IBD [10].

## 3. Problems in Diagnosis of IBD

Despite using all accessible diagnostic methods, the correct diagnosis is not always likely to be established, and the endoscopic and microscopic image is not always certain. Frequently, clinical evaluation do not cover the findings of macro- and microscopic examination. Problems with differential diagnosis of IBD are usually linked to: the effects of chronic inflammation and chronic treatment, failure to find features characteristic for CD (transmural, segmental inflammation, granulomas, deep, linear ulcerations, involvement of small intestine), rapid progress (overlapping of typical features of both UC and CD, macroscopic and microscopic), backwash ileitis – mild inflammation of last few centimeters of ileum, occurring in patients with pancolitis in the course of UC, discreet rectal changes in UC (relative rectal sparing) with the changes more intense proximal to the rectum [55]. Among 10–15% of patients it is impossible to establish final diagnosis, those cases are referred as indeterminate colitis – IC. The diagnosis of IC rises controversies. It may be classified as an autonomic pathoclinical unit, with a different pathogenesis, relating to UC or CD, or as a temporary, introductory diagnosis, which changes after applying all possible diagnostic methods [55]. Discussed problems indicate the need to search for the new diagnostic methods for IBD, including biochemical markers assessing not only the intensity of inflammation, but also enabling to enhance diagnostic accuracy for CD and UC [55].

## 4. Platelet Abnormalities during IBD

Blood platelets are important key regulators in chronic inflammation diseases beyond haemostasis and thrombosis. In IBD pathogenesis, platelet activation could be the missing link between inflammation and coagulation. Platelets are involved in inflammation process by secretion of biologically active substances. These substances are PAF (platelet activation factor), PDGF (platelet-derived growth factor), platelet factor 4, beta-thromboglobulin, fibrinogen, von Willebrand factor (vWF), plasminogen, fibrinolytic inhibitors, coagulation V, VIII and XI factors, protein S, VEGF (vascular endothelial growth factor), P-selectin, ADP, serotonin, IL-1*β*, chemokines, RANTES (regulated on activation, normal T-cell expressed and secreted), IL-8, COX (cyclooxygenase), TF (tissue factor), and PAI-1 (plasminogen activator inhibitor-1) [36, 49, 66].

In IBD many changes in platelet's structure and function occur. Changes such as morphological alterations (MPV, PDW—platelet distribution width, PCT—platelet crit), increase of the count, MPs release, over-excretion of granular content, increased formation of PLT-PLT and PLT-leukocyte aggregates (PLA) are linked with activation of PLT, which is induced by inflammatory agonists. The blood amount of PLT are elevated in patients with increased clinical activity of IBD. This is the effect of incorrect bone marrow thrombopoiesis and reduced PLT lifespan during inflammation [28, 49, 67]. The average size of platelets (MPV) is an important factor of activation of platelets. Large size of platelets indicates their increased enzyme and metabolic activity in inflammation. In Crohn's disease and ulcerative colitis the MPV levels has been found lowest in remission and highest in healthy people. MPV often is decreased in patients with Crohn's disease and ulcerative colitis and it can be a marker of early stages of inflammatory diseases. Inflammation process which occurs in IBD causes an increase of amount of platelets and changes their morphology [37, 49, 68].

In the platelet activation important role plays the secretion of tissue factor from damaged endothelium and the production of thrombin. PAF (platelet activation factor) is a factor secreted by bowel tissue in inflammation (e.g., in IBD) and it also plays role in activation of platelets. These structures secrete proinflammatory mediators such as platelet factor 4, platelet-activating factor, IL-8 and metabolites of arachidonic acid. Biosynthesis of these mediators is increased in patients with IBD. Factors which have properties to diminish platelet activation could prevent the development of thromboembolic complications in patients suffering from IBD [36, 38, 69].

*Beta-thromboglobulin* is an alpha-granule-derived platelet factor which has a higher serum concentration in patients with IBD, but it does not correlate with the activity of disease [20]. It has been also found that the production of the von Willebrand factor, a blood glycoprotein involved in hemostasis which is secreted by platelets, is increased during active IBD [36, 69].

*P-selectin* is an important factor produced by PLT. It also has a soluble fraction detected in patients with inflammatory disease such as IBD. The N-terminal domain of P-selectin binds to PSGL-1 (P-selectin glycoprotein ligand) in leukocytes in the gut mucosa, and initiates processes like chemokine production by monocytes and CD4(+) T cells and superoxide overexcretion by neutrophils. The serum level of soluble platelet selectin (sP-selectin) is increased during the progression of IBD. In inactive Crohn's disease serum levels of sP-selectin are lower than in controls. However, in patients with ulcerative colitis serum concentrations of sP-selectin and IL-6 is significantly higher compared to healthy subjects [36, 49].

Soluble P-selectin is the marker of blood platelet activation, due to its high concentration and its central role in the inflammation process. An increase of sP-selectin concentration occurs with an increasing concentration of IL-6. Moreover, the sP-selectin level in serum shows a positive correlation with PDMPs (platelet-derived microparticles), molecules involved in inflammatory responses. It is an important factor in evaluating the changes of platelet functions in a patient with inflammatory bowel disease. Elevated PDMP levels in patients with active IBD and its significant correlation with sP-selectin suggest that platelets play an important role in the pathogenesis of inflammation [26].

Serum concentration of *VEGF*, which is another factor secreted by platelets in inactive Crohn's, disease is lower than in healthy people [24].

*RANTES* (regulated on activation, normal T-cell expressed and secreted) is one of PLT factors which stimulates polymorphonuclear cells. It enhances production of leukotriene, PAF, and superoxide which is the cause of increased vascular permeability in endothelial cells [41].

In the pathogenesis of venous thromboembolism in patients with inflammatory bowel disease, the increased number of circulating *TF(+) MPs* (procoagulant microparticles) was postulated as an important factor of observed hemostatic abnormalities. It is believed that TF(+) MPs is involved in the pathogenesis of venous thromboembolism in these patients. Circulating TF(+) MPs are a heterogeneous mixture of cellular membrane fragments that are derived from a great variety of cells. Due to the presence of TF and phosphatidylserine on their membrane, they are considered as major procoagulant factors. In patients with IBD, there is a tendency for the formation of thrombin in the vasculature of the intestine and extraintestinal tissues. The increased markers of coagulation are thrombin antithrombin complex, tissue factor, and fibrinopeptide B [33].

Summing up, platelets are key factors in inflammatory diseases. It is observed that IBD increase the number of platelets and production and excretion of granular contents (P-selectin, *β*-TG, PF-4, vWF, and fibrinolytic inhibitors), as well as cause many changes in the platelet's structure and function. The relationship between the factors that play a role in IBD and the risk of cardiovascular disease has been also demonstrated [55].

## 5. The Upcoming Fecal Inflammatory Markers

The main advantage of fecal biomarkers is that these tests measure proteins originating in the intestinal mucosa, which means that they are specific for the bowel and they should reflect intestinal inflammation. Fecal biomarker testing usually has a very high negative predictive value, but its positive predictive value is low. The big advantage of some of the new fecal markers is that they offer a noninvasive, low cost tool which can be used for the prediction of short- and medium-term disease outcomes, including relapse. Markers whose significance in intestinal inflammatory diseases was recently evaluated in multiple research include defensins, beta-glucuronidases, myeloperoxidase, pyruvate kinase, neutrophil gelatinase-associated lipocalin, calgranulin C (S100A12), osteoprotegerin, matrix metalloproteinases, 3-like-chitinase 1, high-mobility nuclear protein, and deoxyribonucleic acid. However, these markers require further evaluation and validation before they can be included in the diagnosis and monitoring of IBD [70] (Table 2).

*Defensins* are peptides acting against microbe infection. *Human defensin β (HBD)* is produced and secreted by intestinal epithelial and plasma cells as a part of the mucosal immune system. The intensity of this process correlates with inflammatory processes in this tissue and can be detected and measured in HBD 2 stool examination. A study conducted among children showed that HBD 2 levels were higher in the control group compared to children with IBD. However in another study, the concentration of HBD 2 was also increased among adults with UC compared to the control group. These results suggest that the HBD 2 marker may not be specific for IBD [70, 71].

*Beta-glucuronidases* are enzymes secreted during inflammatory processes, which can be assessed in the stool specimen. The study analysing the activity of fecal beta-glucuronidase in pediatric patients with IBD, reported that it was 2-fold lower in the IBD group than in the healthy control group [72].

*Myeloperoxidase (MPO)* is an enzyme expressed mainly in granulocytes which release MPO during inflammatory processes. The reaction of MPO with hydrogen peroxide or tyrosine produces highly cytotoxic products that can also contribute to tissue damage in IBD. MPO can be marked in stool examination. The concentration of MPO is significantly statistically increased in patients with active disease and shows a significant correlation with UC. It was also found that the concentration of MPO in feces is significantly statistically higher in patients with UC, compared to patients with CD and to those in the control group. The MPO level in a stool examination has been proved as a noninvasive biomarker for response to treatment in patients with CD and UC [70]. Studies have shown that a high concentration of MPO after treatment suggests an incomplete response to the treatment [73].

*Pyruvate kinase* is an essential enzyme of glycolysis and forms different isozymes: a tetramer (M1) present in the tissue of the brain, skeletal muscle, and heart muscle, and a dimer (M2-PK) which can be useful as a marker. Higher levels of M2-PK can be assessed in feces examination and in serum in the course of inflammatory intestinal diseases and intestinal cancers. The Bcl-xl pathway leading to higher M2-PK expression is one of the possible epithelial antiapoptosis protective mechanisms in CD [70]. The M2-PK protein was found in proliferating cells, such as leukocytes and cancer cells. The concentration of M2-PK in IBD is higher among patients with active disease. Increased M2-PK stool level was also found in pediatric patients with active CD compared to control patients. Pyruvate kinase is a promising sensitive screening tool for intestinal inflammation and its severity indicator [73, 74].

*Neutrophil gelatinase-associated lipocalin (NGAL)*, also known as lipocalin 2, is expressed in inflamed colonic epithelial cells and neutrophilic granulocytes. The mucosal distribution during inflammation makes this protein markedly different from the most studied fecal IBD biomarkers calprotectin and lactoferrin, expressed solely in neutrophilic granulocytes. NGAL is shed to the bowel lumen during active inflammation, causing elevated values in feces. Thus, fecal NGAL *(fe-NGAL)* reflects a different aspect of the inflammatory process than neutrophil infiltration and may represent a more sensitive test than calprotectin in a more chronic inflammatory setting with low numbers of infiltrating granulocytes. Moreover, NGAL act as a growth and differentiation factor and can also stabilize the proteolytic enzyme matrix metalloprotease-9 (MMP-9) [74, 75].

LCN2, the coding gene for NGAL, is one of the most overexpressed genes in the colonic mucosa in UC and CD compared with healthy individuals. The results by Thorsvik et al. and Stallhofer et al. have shown that fe-NGAL was markedly raised in active UC and CD compared with IBS and healthy controls and compared with inactive disease. However, further large-scale studies are needed to elucidate the usefulness of fe-NGAL in the diagnosis of IBD and in the follow-up treatment [74, 75].

*Calgranulin C (S100A12)* is a constitutive cytosolic protein similar to calprotectin, which occurs in human neutrophils, also produced by monocytes. Extracellular S100A12 is secreted by activated or damaged cells under conditions of cellular stress. S100A12 has proinflammatory and chemotactic properties, and it stimulates proinflammatory mediators by NF-kappaB or other similar pathways [76].

Elevated levels of S100A12 are observed in diseases associated with inflammation, for example, rheumatoid arthritis, Kawasaki disease, and cystic fibrosis. High levels of S100A12 are also found in serum and mucosa of the large intestine in children with IBD. In addition, studies have shown that the concentration of S100A12 is elevated in people with irritable bowel syndrome (IBS) [76, 77]. A potential role of this proinflammatory protein has been also proposed in predicting relapse in IBD patients. Fecal concentration of S100/A12 was found significantly higher in the relapse group of patients with CD or UC, in comparison to nonrelapse patients [78].

*Osteoprotegerin (OPG) or osteoclastogenesis inhibitory factor* is a glycoprotein which functions as a receptor preventing the activation of nuclear factor kappa B (NF-*κ*B). The NF-*κ*B is an important inflammation regulator and transcription factor for immune-related genes, and a key regulator of inflammation. The OPG-RANKL complex may contribute to the inflammation of the gastrointestinal mucosa [70]. OPG assessed in a stool specimen was raised in moderate/severe and mild CD, indicating that fecal OPG can be a useful marker of intestinal inflammatory severity in CD [79].

*Matrix metalloproteinases (MMPs)* are a family of key biological mediators involved in the degradation and reconstitution of the extracellular matrix components. Taking into account that MMPs have been proposed to be major factors for intestinal tissue injury mediated by T cells in IBD, an increasing number of studies concentrate on the role of MMPs in IBD [65, 70, 80, 81]. MMPs are expressed in inflammatory areas and ulcerations in the intestine. It has been also proved that MMP-2 takes part in the degradation of the basal membrane type IV collagen and loss of epithelial organisation in active IBD [80]. Gao et al. showed that increased expression of MMP-2 and MMP-9 and mRNA in resected tissue specimens from patients with CD and UC correlates with the severity of inflammation. Immunohistochemical studies showed that MMP-2 was present in the extracellular submucose matrix [76]. In addition, elevated levels of serum and urine MMP-2 were found in patients with IBD. Interestingly, the concentration of MMP-2 in the serum increased, both in patients responding to treatment and in nonresponders [70, 81, 82].

The results by Farkas et al. showed that fecal MMP-9 has high sensitivity in the detection of endoscopically active UC and pouchitis, indicating that the noninvasive method of MMP-9 determination can help assess intestinal inflammation. Moreover, researchers showed that none of the routinely used laboratory activity markers correlates significantly with MMP-9 levels in CD or UC. The level of fecal MMP-9 was the only marker correlating with the clinical symptoms of pouchitis, because it showed a significantly stronger correlation with the activity of UC than with that of CD. Significant correlation was also shown between fecal MMP-9 levels in patients with UC and pouchitis [83].

*3-like-chitinase 1 (CHI3L1)* also called YKL-40, is a glycoprotein which is a heparin, chitin, and collagen-binding member of the mammalian chitinase-like proteins. It is expressed and secreted by different cells, for example, colonic epithelial cells and macrophages. It occurs in an increased amount in the inflammatory mucosa of the large intestine both in vitro and in vivo. Increased CHI3L1 serum concentration has been reported in patients with IBD. Moreover, a significant CHI3L1 increase in stool examination has been shown among children with IBD compared to children in the control group. Chen et al. also associated CHI3L1 with inflammation of colon epithelial cells leading to cancer modification [70]. In CD patients, the stool concentration of CHI3L1 was found to be increased in relation to the control group, whereas in UC the concentration of CHI3L1 in the stool correlated with endoscopic activity with high sensitivity and specificity [73]. Overexpression of CHI3L1 in the large intestine increases the adjacent invasive ability of *Escherichia coli* in the colon. A correlation between CHI3L1 in feces and endoscopic results in a pediatric cohort was also demonstrated [84].

*High-mobility nuclear protein (HMGB)* is a nuclear, nonhistone DNA-binding protein belonging to the HMGB family that consists of 3 nuclear proteins (HMGB1, HMGB2, and HMGB3). The protein consisted of 215 amino acids with 2 tandem DNA-binding domains (80 amino acids for each domain). HMGB1 is a nuclear protein which is involved in maintaining the nucleosome structure and in regulating gene transcription. HMGB1 is released from necrotic cells and can be a different mediator of inflammatory processes. This protein is actively secreted by immune cells such as dendritic cells, neutrophils, monocytes, macrophages, and NK cells. HMGB1 contains 3 conserved cysteines susceptible to redox, C23, C45, and C106, whose modifications in the redox state may also affect their activity outside the cell. When all 3 cysteines are in reduced form, HMGB1 forms a heterocomplex with the chemokine (C-X-C) ligand of the motif 12 (CXCL12), which binds exclusively to CXCR4 to initiate chemotaxis. Oxidation of all 3 cysteines prevents binding between HMGB1 and CXCR4/TLR4, which blocks chemotaxis. HMGB1 protein can be released by cells such as monocytes, macrophages, mature dendritic cells, and natural killer cells into the extracellular milieu to function as a proinflammatory cytokine in response to injury, infection, and inflammation [85].

As a proinflammatory cytokine, it seems to play a role in sepsis, atherosclerosis, chronic kidney disease, cancer, IBD, and other autoimmune diseases such as systemic lupus erythematosus and rheumatic diseases [73]. Studies carried out so far proved that the HMGB1 level assessed in feces examination was significantly higher among children with IBD when compared to the control group. It has been shown that the HMGB1 level is a marker of intestinal inflammation associated with intestinal infection while there is no proven correlation between HMGB1 concentration and CD activity rates [85, 86].

*Deoxyribonucleic acid* is excreted in the stool under physiological conditions among a healthy population while increased levels may indicate disease. DNA and also even more specific *microRNAs* can be marked in the stool as a potential marker in IBD [70]. In addition, the concentration of DNA in the feces of people with UC correlated with its clinical and endoscopic severity, increasing among patients with the relapse of the disease.

Summing up, fecal biomarker testing is currently an evolving field in laboratory medicine with a lot of promise, but we still need more validation and more data showing how these tests could be used to predict a subclinical flare allowing for earlier intervention. New fecal markers may contribute to the improvement of diagnosis, assessment, and clinical outcome of treatment for patients with inflammatory diseases of the intestine.

## 6. The Upcoming Inflammatory Markers Belonging to Extracellular Matrix (ECM) Components

The chronic inflammation which characterized IBD results in excessive turnover of the extracellular matrix. The ECM components are depolymerized into the small fragments, which are released into circulation. The quantitative changes of these fragments into the blood may provide information on the damage of the affected tissue. ECM is composed of fibrous proteins and *glycosaminoglycans (GAGs)* and functions as a dynamic remodeling tissue involved in proliferation, migration, and adhesion. These negatively charged polysaccharides, which regulate the ability of albumin to leave the vasculature and inhibit thrombosis, may be affected by inflammatory cells and their products. Keratan sulfates and dermatan sulfates are sulfated GAG types connected with the intestinal epithelium and regulate its permeability. A substantial loss of sulfated GAGs from the subepithelial basal lamina was found in resected intestinal tissues from IBD patients, both CD and UC, and from the vascular endothelium in submucosa in Crohn's disease [87]. In addition, an increase of GAG degradation pathways has been found in CD. This indicates a connection between ECM homeostasis and inflammatory bowel diseases. Disruption of vascular and connective tissue GAGs, which is related with the inflammatory process could be an important pathogenetic mechanism, contributing to ECM tissue remodeling observed in IBD patients [87].

*Hyaluronan (HA)* is a different nonsulfated linear glycosaminoglycan which greatly affects the integrity of ECM. The simple structure of hyaluronan belies the complexities of its physicochemical properties and biological functions. Among the extracellular matrix (ECM) molecules, it has unique hydrophilic, rheological, and viscoelastic properties. Existing exclusively in the extracellular matrix, HA participates in the forming of the ECM structure [88, 89]. At the cell surface, HA together with extracellular hyaluronan-binding proteins form a hyaluronan-rich multicomponent pericellular matrix, that facilitates cell migration. Another role of HA present at the cell surface is related to its ability to influence signal transduction, a process which is mediated by cell surface hyaluronan receptors, such as CD44, RHAMM, and LEC. Accumulation of HA in the vasculature and elevated HA deposition in the intestinal tissue appear to promote the inflammation process in IBD causing leucocyte infiltration [88–90]. Moreover the serum-derived hyaluronan-associated protein (SHAP-HA) level was found significantly higher in patients with active UC than in those in remission and this value was positively correlated with endoscopic damage. In patients with CD, the serum SHAP-HA level correlated only with TNF-*α* [89].

*Laminin (LN)* is an important component of the intestinal basement membrane, a specialised ECM that is jointly produced by both epithelial and stromal cells. LN as a ubiquitous basement membrane component plays a pivotal role in maintaining the structure and function of basement membranes. Moreover, the local expression of laminin and other tissue extracellular matrix constituents are active participants in the regulation of in situ inflammatory processes during which the structural and functional properties of the local tissue components and of the immune cells themselves are continuously modulated. The LN serum level was found to be higher in CD patients than in controls and it was associated with disease activity [91].

*Syndecan-1 (SDC-1)* is a transmembrane heparan sulfate proteoglycan (HSPG). Intestine-specific HSPGs were found on the basolateral surface of intestinal epithelial cells and have been shown to promote intestinal regeneration, suggesting their role in intestinal stem cell (ISC) homeostasis. Heparan sulfate proteoglycans are one of the most currently studied ECM components in the intestine. Syndecan-1 is the primary heparan sulfate proteoglycan which plays a central role in maintaining normal intestinal barrier function. Moreover, it has been found that shedding of syndecan-1, reflected by soluble syndecan-1 serum concentrations, is highly regulated by inflammation. The SDC-1 levels assessed in the serum of CD patients were found to be higher when compared to the healthy population. In addition, syndecan-1 concentrations were correlated with severity of the disease [92].

*Fibronectin (FN)* is an extracellular glycoprotein which binds with different matrix components. The concentration of fibronectin in the blood was measured in patients with inflammatory bowel disease. Fibronectin levels measured in the blood of patients with CD and UC differentiate from the healthy population. During the process of active inflammation, this marker was significantly lower than in remission. In several cases of CD, the concentration of fibronectin was reduced before clinical relapse and returned to the normal range in remission [93].

It was also found that patients with CD and UC demonstrated autoimmunity to *type VII collagen*. It was concluded that patients with inflammatory bowel disease may have IgG autoantibodies to type VII collagen; however, further studies are required to address the level of this association [94].

## 7. Conclusions

Nowadays, the most accurate diagnosis and differential diagnosis between CD and UC, as well as between IBD and other gastrointestinal diseases, is possible thanks to endoscopic and pathological examination, and none of the laboratory markers is sensitive and specific enough for this purpose [51]. Although considerable progress in the research on candidate biomarkers demonstrating considerable diagnostic and prognostic value and applicable to the clinical setting has been achieved, there is still a long way to go toward the ultimate goal of an ideal biomarker in IBD. Recent data suggest that the performance of the fecal calprotectin test is superior for UC than for CD, but there is urgent necessity for further studies to find a sensitive and noninvasive marker that would undoubtedly differentiate between UC and CD. Combining markers may increase sensitivity and can make disease diagnosis, evaluation of severity, and prognosis more specific. Among several promising markers, reflecting different aspects of IBD response, are new fecal biomarkers which can be used to predict disease and treatment outcomes as well as markers reflecting ECM remodeling related with the inflammatory disease state. ECM components as biomarkers may offer a new tool to evaluate early changes in the gut of patients with IBD, and in time be a good alternative to feces samples or endoscopy. In light of the current intense research, there is an expectation that noninvasive markers useful in differentiating IBD with functional disorders, evaluation of disease activity, prognosis, and treatment selection for IBD will be introduced soon into the clinical practice.

## Figures and Tables

**Table 1:** The most important currently used markers for nonspecific inflammatory bowel diseases (IBD).

Marker	Name	Expression	Comments	References
*Tests evaluating neutrophil activation*				
ENA-78	Epithelial neutrophil activating peptide	Bowel epithelial cells; intestinal epithelial cells	Stimulates the chemotaxis of neutrophils, possesses *angiogenic* properties	[10, 21]
HLE	Human leucocytic elastase	Activated neutrophils	Plays a role in degenerative and inflammatory diseases through proteolysis of collagen-IV and elastin	[6]
MRP-8/MRP-14 or S100A8/A9	Calprotectin	Cytoplasm of neutrophils and monocytes	Antibacterial, antifungal, immunomodulatory, and antiproliferative action; a chemotactic factor for neutrophils; the fecal level is proportional to neutrophilic influx into the intestinal tract	[1, 77]
L	Lactoferrin	Neutrophils	Takes part in acute inflammatory response; exhibits high affinity to iron making iron inaccessible to bacteria; fecal L increases significantly with bowel infiltration by neutrophils	[10, 58]
N	Neopterine	Monocytes and macrophages	Inflammatory marker; may help predict the progress of the disease; useful to assess clinical activity of IBD	[9]
*Serological markers*				
ANCAs	Antineutrophil cytoplasmic antibodies		High p-ANCA levels and antibodies to CBir1 have been associated with increased risk of pouchitis after colectomy in UC	[70]
cANCA	Cytoplasmic	Antibodies against granules of neutrophil cytoplasm	Increase in UC	
sANCA	Speckled		Patients with CD and positive p-ANCA were less likely to respond to therapy with infliximab	
pANCA	Peripheral-antinuclear cytoplasmic antibody		Increase significantly in UC	[74]
ASCAs	Anti-*Saccharomyces cerevisiae* antibodies		The utility in diagnosing difficult cases of indeterminate colitis (IC)	[74]
Anti-OmpC	Antiouter membrane protein C antibody		OmpC pANCA, ASCA, and I2 altogether can be found in 80% of patients with CD	[10]
Hup-B	Mycobacterial histone H1 homologue		May represent the target antigen for pANCA	[10]
Anti-CBir1 flagellin	Antibodies to bacterial flagellin		May be a marker of Crohn's disease complicated by fistulas, perforations, or other serious problems	[10]
PAB	Pancreatic antibody (an antibody to a trypsin-sensitive protein in pancreatic secretions)		PAB is positive in 20%–40% of CD cases and 5% of UC cases; PAB expression may exhibit racial differences	[6]
Anti-I2	Antibodies to *Pseudomonas fluorescens*-associated sequence I2		IgA anti-I2 is positive in 55% of CD cases, 10% of UC cases, and 20% of non-IBD colitis cases; anti-I2 has also been found in patients with other inflammatory enteritis	[10]
*Platelet abnormalities*				
	*β*-Tromboglobulin	Platelets	Increased during active IBD	[20]
	P selectin	Endothelial cells	Serum level of soluble platelet selectin (sP-selectin) is increased during progression of IBD; in inactive CD, serum levels of sP-selectin are lower than in controls; in patients with ulcerative colitis, serum concentrations of sP-selectin and IL-6 are significantly higher compared to healthy subjects	[34, 49]
PAF	Platelet activation factor	Platelets	Increased in patients with IBD	[36]
TF(+) MPs	Procoagulant microparticles	Microparticles circulating into the blood	Indicates for hypercoagulability of blood in IBD patients, which is associated with the appearance of thrombin in the vasculature of the intestine and extraintestinal tissues	[33]

**Table 2:** The novel markers for nonspecific inflammatory bowel diseases (IBD).

Marker	Name	Expression	Comments	References
*Novel fecal markers*				
HBD	Human defensin *β*	Epithelial and plasma cells	Peptides acting against microbe infection, correlate with inflammatory processes	[70]
	*β*-Glucuronidases	Mucosal cells, bacteria	Marker of inflammation	[72]
MPO	Myeloperoxidase	Granulocytes	Marker of inflammation; stool expression higher in patients with UC, compared to patients with CD; biomarker for response to treatment in patients with CD and UC	[70, 73]
M2-PK	Pyruvate kinase	Skeletal muscle, heart, brain, and proliferative tissues	Increases in colorectal carcinoma; in gut, inflammation reflects increased cell turnover; it is postulated that intestinal epithelial cells may be protected against apoptosis by the upregulation of M2-PK in CD; fecal pyruvate kinase has been suggested as a potential new marker for intestinal inflammation in children with IBD and a new predictor for inflammation and severity of pouchitis	[70]
NGAL	Neutrophil gelatinase-associated lipocalin	Ephithelial cells/neutrophilic granulocytes	Contributes to inflammation	[74, 75]
S100A12	Calgranulin C	Neutrophils/macrophages, monocytes	May reflect the presence and severity of intestinal inflammation; has a potential role on predicting relapse	[76, 77]
OPG	Osteoprotegerin	Osteoblasts, B lymphocytes, dendritic cells, bone marrow stromal cells, epithelial cells, and monocytes/macrophages	Useful marker of intestinal inflammatory severity in CD	[70, 79]
MMP	Matrix metalloproteinases	Regenerative tissues	MMPs are expressed in areas of inflammation and ulceration in the gut, and several MMPs are overexpressed in IBD	[70]
CHI3L1	3-Like chitinase	Macrophages, neutrophils, chondrocytes, and synovial cells	Highly expressed in intraepithelial neoplasia mucosa of UC	[70, 84]
HMGB	High-mobility nuclear protein	Neutrophils, monocytes, macrophages, dendritic cells, and natural killer cells	Correlate with disease severity	[77, 85, 86]
DNA	Deoxyribonucleic acid		Fecal excretion of DNA correlates with clinical disease activity and endoscopic severity in UC	[70]
MicroRNA	Microribonucleic acid		Expression patterns have been described in intestinal biopsies collected from IBD patients with a number of specific miRNA reported to be upregulated in both CD and UC	[70]
*Inflammatory markers belonging to extracellular matrix (ECM) components*				
sGAGs	Sulfated glycosaminoglycans	ECM components	Remodeling tissue involved in proliferation, migration and adhesion;	[87]
HA	Hyaluronian	Nonsulfated GAG; ECM component	Elevated HA deposition in the intestine tissue promotes inflammation in IBD	[88–90]
LN	Laminin	Basement membrane component	LN serum level is higher in CD than in controls and it is associated with disease activity	[91]
SDC-1	Syndecan-1	Transmembrane heparan sulfate proteoglycan	Inflammatory marker; soluble SDC-1 levels are higher in CD patients and may contribute to the assessment of disease activity	[92]
FN	Fibronectin	ECM component	In several cases of CD, the concentration of fibronectin in the blood plasma was reduced before clinical relapse and returned to the normal range in remission	[93]
COLVII-Ab	Autoantibodies against type VII collagen	Tissues with high expression of collagen VII, including colonic epithelium	CD and UC demonstrated reactivity to type VII collagen	[94]
